# The Physician’s Interpreter

**Published:** 1888-10

**Authors:** 


**The Physician's Interpreter.** In four languages. By M. VON V. Philadelphia: F. A. Davis, 1231 Filbert street.

An attempt is made in this little book to frame such questions as will aid in diagnosis, adapting them to the English,

French, German and Italian languages. Theoretically this plan ought to be of value to such physicians and students as already have some knowledge of the pronunciation of foreign languages; it must, however, be tested as to its practical value before this can be determined.

